# Immunotherapy in People With HIV and Cancer

**DOI:** 10.3389/fimmu.2019.02060

**Published:** 2019-08-28

**Authors:** Camille E. Puronen, Emily S. Ford, Thomas S. Uldrick

**Affiliations:** ^1^Division of Medical Oncology, Department of Medicine, University of Washington, Seattle, WA, United States; ^2^Division of Infectious Diseases, Department of Medicine, University of Washington, Seattle, WA, United States; ^3^Vaccine and Infectious Diseases Division, Fred Hutchinson Cancer Research Center, Seattle, WA, United States; ^4^Division of Global Oncology, Department of Medicine, Fred Hutchinson Cancer Research Center, Seattle, WA, United States

**Keywords:** HIV, immunotherapy, HIV reservoir, cancer, PD-1, Kaposi sarcoma, lymphoma

## Abstract

HIV infection alters the natural history of several cancers, in large part due to its effect on the immune system. Immune function in people living with HIV may vary from normal to highly dysfunctional and is largely dependent on the timing of initiation (and continuation) of effective antiretroviral therapy (ART). An individual's level of immune function in turn affects their cancer risk, management, and outcomes. HIV-associated lymphocytopenia and immune dysregulation permit immune evasion of oncogenic viruses and premalignant lesions and are associated with inferior outcomes in people with established cancers. Various types of immunotherapy, including monoclonal antibodies, interferon, cytokines, immunomodulatory drugs, allogeneic hematopoietic stem cell transplant, and most importantly ART have shown efficacy in HIV-related cancer. Emerging data suggest that checkpoint inhibitors targeting the PD-1/PD-L1 pathway can be safe and effective in people with HIV and cancer. Furthermore, some cancer immunotherapies may also affect HIV persistence by influencing HIV latency and HIV-specific immunity. Studying immunotherapy in people with HIV and cancer will advance clinical care of all people living with HIV and presents a unique opportunity to gain insight into mechanisms for HIV eradication.

## Introduction

People living with HIV (PLWH) have an elevated risk of developing cancer compared to the general population. This increased risk is partially attributable to comorbid conditions and social factors such as smoking or poorer access to preventative services. However, there is strong evidence that immunologic factors such as decreased immunologic surveillance and increased susceptibility to oncogenic viral infection play a significant role (1–5). Historically, cancers developing in the setting of HIV have been classified as AIDS-defining malignancies (ADM; cancers that, when present, confer a diagnosis of AIDS) and non-AIDS defining malignancies (NADM; cancers whose presence does not necessarily indicate AIDS) (6). Many HIV-related cancers have a viral etiology (7). These include Kaposi sarcoma (KS) [Kaposi sarcoma herpes virus (KSHV)]; cervical, anal, penile and vulvar squamous cell cancer and oropharyngeal cancers [human papilloma virus (HPV)]; B cell non-Hodgkin lymphomas (NHL) including diffuse large B-cell lymphoma, Burkitt lymphoma, plasmablastic lymphoma, primary central nervous system lymphoma, primary effusion lymphoma, classic Hodgkin lymphoma, and lymphoproliferative disorders [in some cases, Epstein-Barr virus (EBV) and/or KSHV]; hepatocellular carcinoma [hepatitis B and C viruses (HBV/HCV)], and Merkel cell carcinoma [Merkel cell polyoma virus (MPV)]. In epidemiological studies of non-Hodgkin lymphoma, Kaposi sarcoma, and anal cancer, uncontrolled HIV viremia is an independent risk factor (4, 5, 8).

The introduction of antiretroviral therapy (ART) after 1996 resulted in a reduction in the incidence of many ADMs by 75–80% (9), largely due to reduced prevalence of profound immunodeficiency. NADMs including lung cancer, Hodgkin lymphoma, anal cancer, and oropharyngeal cancer now comprise an increasing proportion of total cancers in PLWH in North America (10, 11). A similar trend has been documented in Europe, Australia (12) and the Asia-Pacific region (11, 13). This epidemiological switch in prevalence away from ADMs and virally-associated malignancies corresponds with increasing life expectancy of PLWH, increased availability of ART and promotion of viral suppression (14–16).

## HIV Leads to Partially Reversable Perturbation in T-Cell Function

HIV has multiple effects on T-cell immunity that may contribute to cancer risk. Absent effective ART, uncontrolled HIV infection leads to massive depletion of HIV-infected CD4 cells and uninfected bystander CD4s in both blood and tissue (17). In the same setting, CD8 counts often rise, leading to inverted CD4/CD8 ratios that are an independent measure of immune dysfunction. Moreover, HIV and other chronic viral infections lead to increased expression of immune checkpoint proteins (such as PD-1), exhaustion markers, and impaired CD8 T cell function (18–20), causing systemic immune dysfunction and dysregulation (21). Untreated HIV perturbs not only the quantity but also the breadth of T-cell immunity. HIV leads to decreased numbers of naïve T cells, less diversity of the T-cell repertoire in the blood (22, 23), and skewing of the T-cell receptor (TCR) repertoire secondary to CD4 depletion and expansion of oligoclonal CD8 populations (24). HIV viremia is rapidly suppressed with modern ART. Immune reconstitution after initiation of ART leads to CD4 recovery and CD8 decline over time (25). The likelihood of full immune recovery improves with earlier diagnosis and a younger age at ART initiation (26), although immune recovery is often incomplete (27). The heightened pro-inflammatory state associated with both untreated and treated HIV contributes to long-term adverse outcomes (28, 29).

## Oncogenesis in the Setting of HIV-Induced Immune Dysfunction

Immunodeficiency is an established risk factor for the development of cancer, and the underlying causes are likely many, including uncontrolled proliferation of oncogenic viruses and inadequate immune surveillance. Many oncogenic viruses have been shown to cause cancer in other immunosuppressed states, including inherited immunodeficiencies and solid-organ transplantation (30). CD4 deficiency is strongly linked to malignancy (31), independent of HIV infection (32–35). The presence, number, and functionality of CD4 T cells are important in multiple steps of the oncogenic pathway, including recognition of tumor antigens, development of effective neutralizing antibody, and cellular responses to viral pathogens, and clearance of premalignant lesions. The risk of many HIV-associated malignancies decreases with improved CD4 count on ART (9, 12, 36–39) and cancer-specific mortality correlates inversely with CD4 count (12, 40). The link between reduced CD4 count and elevated cancer risk is profound in KS and NHL (41–43), but also present in other malignancies (37). An individual's risk of cancer (and long-term immune dysfunction) is likely influenced by the CD4 nadir, perhaps indicative of a synergistic relationship between chronic inflammation and impaired immune surveillance (10, 44–49).

CD4 lymphocytopenia, ineffective CD8 response, and associated immune dysregulation lead to a reduction in immunosurveillance, a key mechanism in HIV-associated oncogenesis (21, 50). This is illustrated in the link between HIV, immune status, and cervical cancer (37). PLWH are more likely to acquire high risk HPV (51, 52), less likely to clear HPV, and more likely to progress to higher-grade forms of dysplasia (53). PLWH with lower CD4 counts are also more likely to progress from dysplasia to invasive cancer (54). In an HPV vaccine trial in adolescents with HIV, the induced antibody titer correlated positively with CD4 count (55), supporting the importance of CD4 T cells in the production of high-affinity antibodies (51), the primary correlate of protection of the HPV vaccine (56). Tissue-localizing HPV-specific CD4 and CD8 T cells are also potentially important to tumor regression (57, 58).

Immune exhaustion and T-cell senescence are prominent features of both chronic viral infections and malignancies (59). In PLWH, T-cell dysfunction is most strongly implicated in the development of EBV-related lymphomas and KS (60). In HIV-associated B cell NHL, reduced T-cell polyfunctionality and TCR diversity is associated with poorer prognosis (61). These observations, among others (62), have led to interest in remedying immune dysfunction to treat malignancy in PLWH (63).

## Antiretroviral Therapy and Other Forms of Immunotherapy in HIV-Related Cancer

ART is itself an effective form of immunotherapy for ADM. Improvements in ART in 1996 resulted in a decline in the incidence and severity of KS, as well as changes in its natural history (9, 64–66): the risk of death due to KS decreased at similar HIV RNA levels and CD4 count (66), suggesting that ART resulted not only in improved immune control of KSHV but also decreased immune dysregulation. ART-induced immune reconstitution results in regression of KS lesions in ~80% of PLWH with early KS (67). However, ART alone is often insufficient in advanced KS.

Several immunotherapies have shown efficacy in KS and other HIV-related cancers (Table 1). Interferon alpha (IFN-α), the first true immunotherapy used in HIV-associated KS, generated a 20–40% response rate (98–100). IL-12, which enhances Th-1 type immune responses (91), has been shown to have anti-KS activity in patients who are progressing despite ART (92) and is currently being developed as a tumor-targeted immunocytokine, NHS-IL12 (101). A recent trial of the immunomodulatory drug pomalidomide in 22 participants with heavily pretreated KS who were virally suppressed on ART noted an overall response rate of 60% among HIV-infected participants, which is comparable to traditional cytotoxic chemotherapy for KS. The investigators observed expansion of central memory cells and decreases in CD57+ immunosenescent T-cells (73, 74).

**Table 1:** Select immunotherapeutic agents used in cancers that occur at increased frequency in people with HIV and their demonstrated or hypothesized effect on measurements of the HIV reservoir.

**Agent**	**Mechanism**	**Indication in cancer that is associated with HIV**	**Adverse events**	**Potential effect on HIV reservoir**	**References**
Checkpoint inhibitors (ipilimumab nivolumab, pembrolizumab, durvalumab, etc.)	Block inhibitory T cell receptors including CTLA4, PD-1, or PD-L1, allowing T cell activation and promoting cytotoxic killing of target cells	Lung cancer, classical Hodgkin lymphoma, head and neck cancer, liver cancer	Fatigue, rash, arthralgia, pruritis, GI toxicity, asthenia, pulmonary toxicity, pyrexia, autoimmune phenomena, headache	Transient increases in unspliced HIV RNA and decreases in HIV DNA in blood, variable effects on plasma HIV RNA	(68–72)
Pomalidomide	Modulates substrate specificity of cereblon E3 Ubiquitin ligase, altering protein expression. Induces cell cycle arrest and apoptosis in plasma cell malignancies. Enhances T cell- and natural killer (NK) cell-mediated cytotoxicity, inhibits angiogenesis, modulates cytokines, and cell microenvironment	Under evaluation for KS	Thromboembolic events, teratogenicity, fatigue and asthenia, cytopenias, GI toxicity, dyspnea, back pain, pyrexia	Immune stimulation, increased killing of reservoir cells	(73–75)
Brentuximab vedotin	Monoclonal antibody drug conjugate with anti-CD30 antibody (expressed on Hodgkin Reed-Sternberg Cells) and MME (microtubule disruptor) payload	Classical Hodgkin lymphoma	Cytopenias, peripheral sensory neuropathy, fatigue, GI toxicity, pyrexia, rash, cough	Transient loss of detectable CD4 T-cell HIV RNA and reduction in plasma HIV viremia	(76, 77)
Alemtuzumab	Monoclonal antibody to CD52 (expressed on lymphocytes, monocytes, macrophages, NK cells, and some granulocytes)	Hematopoietic stem cell transplant conditioning	Infusion reaction, serious infections, cytopenias, secondary autoimmune disorders	*Ex vivo* elimination of latently-infected CD4 T cells. Evidence of decreased frequency of HIV-infected CD4 T cells *in vivo*.	(78–81)
IL-7	Modulates T cell development and maturation in the thymus. Modulates T cell homeostasis and proliferation and memory differentiation. Inhibits T cell apoptosis and promotes proliferation.	Under evaluation in combination with CD19 CAR T-cells in relapsed B-cell lymphoma	Infusion reaction, hypersensitivity	Transient increases in HIV viral load without observed clinical sequelae, as well as enhanced anti-HIV CD8 activity	(82–90)
IL-12	Promotes activation and differentiation of T lymphocytes and NK cells	Under evaluation in therapeutic vaccines for HPV associated cancers, phase 1 studies in solid tumors.	Immune activation	Latency reversal *ex vivo*	(91–93)
IL-15	Stimulates the proliferation of memory T cells and regulates their turnover. Promotes the survival of naive T cells.	Under evaluation in refractory B-cell lymphomas and solid tumors	Infusion reaction, hypersensitivity	*Ex vivo* killing of latently-infected CD4 T cells by cytotoxic CD8 T cells	(94–97)

Despite immune dysfunction due to HIV, cancer in PLWH is often responsive to immunotherapy. Thus far, the best-studied agents are tumor-targeting monoclonal antibodies in the management of HIV-associated lymphomas. Rituximab, a monoclonal antibody to the B-cell antigen CD20 that works in part through antibody-dependent cell-mediated cytotoxicity, is associated with improved overall survival in NHL when compared to chemotherapy alone (102–104). In people with HIV-associated lymphoma, a pooled analysis of over 1,500 patients noted that rituximab improved overall survival in those with a CD4 count >50 cells/μL (105). Brentuximab vedotin, an antibody-drug conjugate directed at CD30 on Reed-Sternberg cells, has been shown to have activity in HIV-associated Hodgkin lymphoma: in a study of 6 patients with HIV and classical Hodgkin lymphoma, all achieved a complete response with minimal hematologic toxicity or infectious complications (106).

More recently, immune checkpoint inhibitors (CPIs), monoclonal antibodies to cytotoxic lymphocyte associated protein 4 (CTLA-4) or programmed cell death 1 or its ligand (PD-1 and PD-L1), have gained widespread use due to their demonstrated activity and favorable toxicity profile in many malignancies. CPIs, which function by blocking T-cell inhibitory signaling, have performed well in clinical trials of many malignancies that are common in the setting of HIV, including lymphoma, lung cancer, cervical cancer, liver cancer, and head and neck cancers (107, 108). While nearly all these trials excluded PLWH (109), case reports and retrospective cohort studies from US and European collaborative groups have described an acceptable safety profile with the use of nivolumab, pembrolizumab, and ipilimumab in PLWH, with reported tumor responses in classical Hodgkin lymphoma, melanoma and lung cancer (68, 69, 110–116). A systematic review of CPIs in PLWH noted overall response and adverse event rates that were similar to the general population. In the subset of patients in whom viral load was measured, HIV remained suppressed in 93% of participants, and CD4 counts increased modestly. Notably, CPI use in KS was associated with an overall response rate of 63% (117). A prospective cohort study of 10 PLWH with NSCLC treated with nivolumab noted similar response rates to HIV-uninfected patients: 2 patients had a partial response, 4 had stable disease, and 4 progressed. All patients tolerated nivolumab well with no serious adverse events (70). A prospective phase 1 study of pembrolizumab in PLWH with a CD4 count >100 cells/μl and advanced cancer demonstrated evidence of safety and activity in KS, NHL, lung cancer, and liver cancer (118). A study of durvalumab in 20 aviremic PLWH with advanced solid tumors likewise reported no serious adverse events, nor evidence of HIV reactivation during durvalumab therapy (119). Ongoing studies evaluating CPIs in HIV-associated cancers include a phase 1 study of nivolumab (anti-PD-1) combined with ipilimumab (anti-CTLA-4) in relapsed classical Hodgkin lymphoma or solid tumors (NCT02408861), a phase 2 study of nivolumab in advanced non-small cell lung cancer (NCT03304093), a phase 2 study of durvalumab in advanced cancer (NCT03094286), a study of pembrolizumab as first systemic therapy in KS (NCT02595866), and intralesional nivolumab for limited cutaneous KS (NCT03316274).

## Cancer Immunotherapy and HIV Persistance

**Figure 1:**
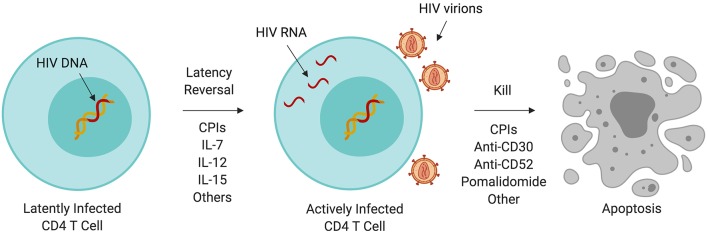
Immunotherapy and the HIV reservoir. A variety of immunotherapeutic agents used to treat cancer may perturb the HIV reservoir through induction of latency reversal or increased cell killing. Some of these agents are being evaluated in clinical trials targeting HIV persistence. CPI, immune checkpoint inhibitor; IL, interleukin.

Although HIV-infected individuals on ART may have undetectable plasma HIV RNA by standard clinical assays, a reservoir of latently HIV-infected cells (120, 121) persists from which the virus will resurface after discontinuation of ART (122). Persistence of the HIV reservoir is partly due to the inherent longevity of resting memory CD4 T cells; growing evidence suggests that its persistence is maintained by clonal expansion (123, 124). In whole genome-based studies, HIV integration favors sites of active gene transcription (125) which benefits HIV replication and establishment of latency (126, 127) and promotes pathways associated with oncogenesis (124). The HIV reservoir has been a major subject of research into a functional cure for HIV. One theory called “kick and kill” (Figure 1) (128, 129) proposes that HIV latency reversal in the setting of ART (meaning activation of HIV replication within a latently infected cell), can lead to increased immunogenicity of HIV infected cells, enhancement of anti-HIV immunity, and increased cell death of HIV reservoir cells.

Several immunotherapeutic agents used in the treatment of cancer may have cause HIV latency reversal and/or have a targeted effect on HIV persistence. CPIs have been proposed to have latency reversal activity. Anti-PD-1 therapy is associated with changes in CD4 count and HIV RNA (130–132), perhaps due to direct targeting of the HIV reservoir. PD-1 and CTLA-4 expression are increased in the setting of chronic HIV infection, and HIV DNA and unspliced RNA are enriched in PD-1+ cells in blood and lymph nodes of individuals with HIV on ART (133–136). Multiple case reports and prospective studies have documented transient increases in HIV transcription in CD4 cells in people with HIV-associated malignancies on ART who are treated with anti-PD-(L)1 drugs, although many of these participants later experienced decreases in plasma HIV RNA (117, 128, 129, 132, 137). In one study, 2 of 28 patients who had undetectable HIV RNA prior to CPI therapy developed detectable HIV RNA, whereas 5 of 6 patients who had detectable viremia experienced a decrease in their viral load (117). A prospective study of the effect of ipilimumab in 24 PLWH with detectable viremia and without cancer, of whom 17 were on ART, also demonstrated a range of responses: 2 participants had slight decreases in HIV RNA but 14 had slight increases. None experienced significant change in CD4 or CD8 T cell count (138). These observations support the activity of CPIs to produce latency reversal. Additional studies are being performed to evaluate the effects of CPIs on anti-HIV T-cell function.

The effects of anti-CD30 monoclonal antibodies on HIV latency have also been investigated. Early work in HIV demonstrated that cross-linking of CD30 on latently-infected CD4 T cells induced HIV transcription (139). More recently, brentuximab vedotin has been associated with transient loss of detectable CD4 T-cell HIV RNA and reduction in plasma HIV viremia (76). CD30 is therefore speculated to be a marker of latent, but transcriptionally-active, HIV-infected cells and a potential therapeutic target for HIV eradication (140).

Alemtuzumab is a monoclonal antibody targeting CD52, which is expressed by T cells including HIV-infected T cells, regardless of CD4 count or plasma viremia. Latently-infected CD4 T cells have been eliminated *in vitro* with alemtuzumab (78). *In vivo*, a case report of alemtuzumab in an individual with HIV and Sezary syndrome described decreased frequency but not elimination of HIV-infected CD4 T cells (79). Alemtuzumab was also part of the conditioning regimen of one of the patients with sustained HIV aviremia after HSCT (141).

T-cell growth factors, many of which are being investigated for cancer indications, have also been shown to affect the HIV reservoir. Interleukin 7 (IL-7) is a homeostatic cytokine that increases T-cell repertoire diversity through expansion of naive T cells (82) and is being investigated in several malignancies. IL-7 levels increase in HIV-associated CD4 lymphocytopenia and decrease with immune reconstitution (142). Exogenous administration of IL-7 is associated with dose-dependent increases in CD4 and CD8 T cells in PLWH on ART (143), including HIV-specific CD8 T cells (83). In patients with suppressed HIV, administration of IL-7 led to transient increases in HIV viral load without observed clinical sequelae (84), as well as enhanced anti-HIV CD8 activity. Another T-cell growth factor, IL-15, induces antigen-specific T-cell proliferation, most pronounced in the CD8 compartment (94, 95, 144, 145). IL-15 is produced during acute HIV infection (95). Stimulating NK cells with IL-15 *ex vivo* from participants with suppressed HIV on ART led to *ex vivo* killing of latently-infected CD4 T cells by cytotoxic CD8 T cells (96). Early phase studies of IL-7 and IL-15 in several malignancies are underway.

## Hematopoietic Stem Cell Transplantation in HIV

In 2007, an individual with HIV infection and leukemia underwent hematopoietic stem cell transplant (HSCT) in Berlin, using cells from a donor who was homozygous for CCR5-delta32, a mutation that renders CD4 cells resistant to CCR5-tropic HIV. After transplant, HIV was undetectable in blood and biopsy specimens, despite discontinuation of ART (146, 147). Recently, a second patient who underwent allogeneic HSCT for Hodgkin lymphoma using cells from a homozygous CCR5-delta32 donor and whose HIV remained undetectable 18 months after stopping ART (141) was described. Allogeneic stem cell transplant itself appears to substantially decrease the HIV reservoir. In the European IciStem cohort of PLWH on ART who underwent HSCT for hematologic malignancies from CCR5 wild-type donors with full donor engraftment and who remained on ART, 5 of 6 were found to have no detectable HIV DNA in CD4 cells from blood and tissues and no evidence of HIV in a humanized mouse viral outgrowth assay (148). However, ART interruption is required to demonstrate functional cure, and in cases of allotransplants from CCR5 wild-type donors, HSCT has failed to produce long-lasting viral suppression in the absence of ART. In an ART interruption study of 2 PLWH who underwent HSCT for hematologic malignancies from CCR5 wild-type donors and had undetectable HIV RNA for years post-transplant while on ART, both participants developed detectable viremia after ART interruption: patient A at day 84 and patient B at day 225 (149).

Given the success of allotransplants from homozygous CCR5-delta32 donors, CCR5-mutant cell products have been developed via gene editing and have been shown to be safe when infused into participants with chronic aviremic HIV. When ART was interrupted, the edited CD4 cells declined at a slower rate than endogenous CD4 cells. While these results are promising, additional work is required to develop a scalable approach to address HIV persistence on ART (150–153).

## Improving Our Understanding of HIV-Related Cancer

As PLWH are living longer, cancer has become a major cause of morbidity and mortality, well above the burden faced by the general population. Although the incidence of AIDS-defining malignancies has decreased, mortality associated with NADMs is rising. Given the persistent immune abnormalities despite ART and the implications for cancer risk, immunotherapy is uniquely poised to improve outcomes in HIV-associated cancers. In order to advance our understanding, PLWH must be included in immuno-oncology studies. Recent recommendations from ASCO and the FDA provide guidance for appropriate inclusion of PLWH and cancer in clinical trials (109, 154). Furthermore, studying cancer immunotherapy in this population represents an opportunity to gain a better understanding of HIV itself. Investigation of the immunologic and viral responses to cancer immunotherapy in PLWH will lead to novel insights into HIV elimination and, above all, improve the outcomes of people with HIV and cancer.

## Author Contributions

All authors listed have made a substantial, direct and intellectual contribution to the work, and approved it for publication.

### Conflict of Interest Statement

TU reports research support for investigator initiated research from Merck, Celgene, and Roche outside the submitted work. In addition TU has a patent 10,001,483 B2 issued. The remaining authors declare that the research was conducted in the absence of any commercial or financial relationships that could be construed as a potential conflict of interest.

## References

[1] Coghill AE Han X Suneja G Lin CC Jemal A Shiels MS. Advanced stage at diagnosis and elevated mortality among US patients with cancer infected with HIV in the National Cancer Data Base. Cancer. (2019) 125:2868–76. 10.1002/cncr.32158 31050361 PMC6663596

[2] Shepherd L Ryom L Law M Petoumenos K Hatleberg CI d'Arminio Monforte A. Cessation of cigarette smoking and the impact on cancer incidence in human immunodeficiency virus-infected persons: the data collection on adverse events of anti-HIV drugs study. Clin Infect Dis. (2019) 68:650–7. 10.1093/cid/ciy508 29912335

[3] Grover S Desir F Jing Y Bhatia RK Trifiletti DM Swisher-McClure S. Reduced cancer survival among adults with HIV and AIDS-defining illnesses despite no difference in cancer stage at diagnosis. J Acquir Immune Defic Syndr. (2018) 79:421–9. 10.1097/QAI.0000000000001842 30211722 PMC6203623

[4] Hernandez-Ramirez RU Qin L Lin H Leyden W Neugebauer RS Althoff KN Association of immunosuppression and HIV viremia with anal cancer risk in persons living with HIV in the United States and Canada. Clin Infect Dis. (2019) 2019:ciz329 10.1093/cid/ciz329 PMC7319056 31044245

[5] Hernández-Ramírez RU Qin L Lin H Leyden W Neugebauer RS Althoff KN. Association of immunosuppression and HIV viraemia with non-Hodgkin lymphoma risk overall and by subtype in people living with HIV in Canada and the USA: a multicentre cohort study. Lancet HIV. (2019) 6:e240–e9. 30826282 10.1016/S2352-3018(18)30360-6 PMC6531288

[6] Patel P Hanson DL Sullivan PS Novak RM Moorman AC Tong TC. Incidence of types of cancer among HIV-infected persons compared with the general population in the United States, 1992–2003. Ann Intern Med. (2008) 148:728–36. 10.7326/0003-4819-148-10-200805200-00005 18490686

[7] Park LS Hernández-Ramírez RU Silverberg MJ Crothers K Dubrow R. Prevalence of non-HIV cancer risk factors in persons living with HIV/AIDS: a meta-analysis. AIDS. (2016) 30:273–91. 10.1097/QAD.0000000000000922 26691548 PMC4689318

[8] Achenbach CJ Buchanan AL Cole SR Hou L Mugavero MJ Crane HM. HIV viremia and incidence of non-Hodgkin lymphoma in patients successfully treated with antiretroviral therapy. Clin Infect Dis. (2014) 58:1599–606. 10.1093/cid/ciu076 24523217 PMC4017888

[9] Franceschi S Lise M Clifford GM Rickenbach M Levi F Maspoli M. Changing patterns of cancer incidence in the early- and late-HAART periods: the Swiss HIV Cohort Study. Br J Cancer. (2010) 103:416–22. 10.1038/sj.bjc.6605756 20588274 PMC2920013

[10] Franzetti M Ricci E Bonfanti P. The pattern of non-AIDS-defining cancers in the HIV population: epidemiology, risk factors and prognosis. A review. Curr HIV Res. (2019) 17:1–12. 10.2174/1570162X17666190327153038 30919779

[11] Nagata N Nishijima T Niikura R Yokoyama T Matsushita Y Watanabe K. Increased risk of non-AIDS-defining cancers in Asian HIV-infected patients: a long-term cohort study. BMC Cancer (2018) 18:1066. 10.1186/s12885-018-4963-8 30400779 PMC6219071

[12] Worm SW Bower M Reiss P Bonnet F Law M Fätkenheuer G. Non-AIDS defining cancers in the D:A:D Study–time trends and predictors of survival: a cohort study. BMC Infect Dis. (2013) 13:471. 10.1186/1471-2334-13-471 24106926 PMC3852673

[13] Jung IY Rupasinghe D Woolley I O'Connor CC Giles M Azwa RI. Trends in mortality among ART-treated HIV-infected adults in the Asia-Pacific region between 1999 and 2017: results from the TREAT Asia HIV Observational Database (TAHOD) and Australian HIV Observational Database (AHOD) of IeDEA Asia-Pacific. J Int AIDS Soc. (2019) 22:e25219. 10.1002/jia2.25219 30615271 PMC6322485

[14] Shiels MS Pfeiffer RM Gail MH Hall HI Li J Chaturvedi AK. Cancer burden in the HIV-infected population in the United States. J Natl Cancer Inst. (2011) 103:753–62. 10.1093/jnci/djr076 21483021 PMC3086877

[15] Cobucci RN Lima PH de Souza PC Costa VV Cornetta Mda C Fernandes JV. Assessing the impact of HAART on the incidence of defining and non-defining AIDS cancers among patients with HIV/AIDS: a systematic review. J Infect Public Health. (2015) 8:1–10. 10.1016/j.jiph.2014.08.003 25294086

[16] Coghill AE Shiels MS Suneja G Engels EA. Elevated cancer-specific mortality among HIV-infected patients in the United States. J Clin Oncol. (2015) 33:2376–83. 10.1200/JCO.2014.59.5967 26077242 PMC4500831

[17] Doitsh G Cavrois M Lassen KG Zepeda O Yang Z Santiago ML. Abortive HIV infection mediates CD4 T cell depletion and inflammation in human lymphoid tissue. Cell. (2010) 143:789–801. 10.1016/j.cell.2010.11.001 21111238 PMC3026834

[18] Cockerham LR Jain V Sinclair E Glidden DV Hartogenesis W Hatano H. Programmed death-1 expression on CD4? and CD8? T cells in treated and untreated HIV disease. AIDS. (2014) 28:1749–58. 10.1097/QAD.0000000000000314 24871455 PMC4206412

[19] Khoury G Fromentin R Solomon A Hartogensis W Killian M Hoh R. Human immunodeficiency virus persistence and T-cell activation in blood, rectal, and lymph node tissue in human immunodeficiency virus-infected individuals receiving suppressive antiretroviral therapy. J Infect Dis. (2017) 215:911–9. 10.1093/infdis/jix039 28453847 PMC5407052

[20] Betts MR Nason MC West SM De Rosa SC Migueles SA Abraham J. HIV nonprogressors preferentially maintain highly functional HIV-specific CD8+ T cells. Blood. (2006) 107:4781–9. 10.1182/blood-2005-12-4818 16467198 PMC1895811

[21] Porichis F Hart MG Massa A Everett HL Morou A Richard J. Immune checkpoint blockade restores HIV-specific CD4 T cell help for NK cells. J Immunol. (2018) 201:971–81. 10.4049/jimmunol.1701551 29934472 PMC6064609

[22] Baum PD Young JJ Schmidt D Zhang Q Hoh R Busch M. Blood T-cell receptor diversity decreases during the course of HIV infection, but the potential for a diverse repertoire persists. Blood. (2012) 119:3469–77. 10.1182/blood-2011-11-395384 22371879 PMC3325037

[23] Connors M Kovacs JA Krevat S Gea-Banacloche JC Sneller MC Flanigan M HIV infection induces changes in CD4+ T-cell phenotype and depletions within the CD4+ T-cell repertoire that are not immediately restored by antiviral or immune-based therapies. Nat Med. (1997) 3:533–40. 10.1038/nm0597-533 9142122

[24] Heather JM Best K Oakes T Gray ER Roe JK Thomas N. Dynamic Perturbations of the T-cell receptor repertoire in chronic HIV infection and following antiretroviral therapy. Front Immunol. (2015) 6:644. 10.3389/fimmu.2015.00644 26793190 PMC4707277

[25] Funderburg NT Andrade A Chan ES Rosenkranz SL Lu D Clagett B. Dynamics of immune reconstitution and activation markers in HIV+ treatment-naive patients treated with raltegravir, tenofovir disoproxil fumarate and emtricitabine. PLoS ONE. (2013) 8:e83514. 10.1371/journal.pone.0083514 24367599 PMC3867440

[26] Wilson EM Sereti I Immune restoration after antiretroviral therapy: the pitfalls of hasty or incomplete repairs. Immunol Rev. (2013) 254:343–54. 10.1111/imr.12064 23772630 PMC3694599

[27] Kelley CF Kitchen CM Hunt PW Rodriguez B Hecht FM Kitahata M. Incomplete peripheral CD4+ cell count restoration in HIV-infected patients receiving long-term antiretroviral treatment. Clin Infect Dis. (2009) 48:787–94. 10.1086/597093 19193107 PMC2720023

[28] Castillo-Mancilla JR Phillips AN Neaton JD Neuhaus J Sharma S Baker JV. Incomplete ART adherence is associated with higher inflammation in individuals who achieved virologic suppression in the START study. J Int AIDS Soc. (2019) 22:e25297. 10.1002/jia2.25297 31250552 PMC6597899

[29] Nasi M De Biasi S Gibellini L Bianchini E Pecorini S Bacca V. Ageing and inflammation in patients with HIV infection. Clin Exp Immunol. (2017) 187:44–52. 10.1111/cei.12814 27198731 PMC5167025

[30] Bonnet F Lewden C May T Heripret L Jougla E Bevilacqua S. Malignancy-related causes of death in human immunodeficiency virus-infected patients in the era of highly active antiretroviral therapy. Cancer. (2004) 101:317–24. 10.1002/cncr.20354 15241829

[31] Clifford GM Franceschi S. Cancer risk in HIV-infected persons: influence of CD4+ count. Future Oncol. (2009) 5:669–78. 10.2217/fon.09.28 19519206

[32] Dutta A Uno H Lorenz DR Wolinsky SM Gabuzda D. Low T-cell subsets prior to development of virus-associated cancer in HIV-seronegative men who have sex with men. Cancer Causes Control. (2018) 29:1131–42. 10.1007/s10552-018-1090-4 30315476 PMC6245112

[33] Grossman D Lewis DE Ballas ZK Duvic M. Idiopathic CD4+ T lymphocytopenia in a patient with mycosis fungoides. J Am Acad Dermatol. (1994) 31:275–6. 10.1016/S0190-9622(08)81978-0 8040416

[34] Paolini R D'Andrea E Poletti A Del Mistro A Zerbinati P Girolami A. B Non-Hodgkin's Lymphoma in a Haemophilia Patient with Idiopathic CD4+ T-lymphocytopenia. Leuk Lymph. (1996) 21:177–80. 10.3109/10428199609067597 8907287

[35] Menetrier-Caux C Ray-Coquard I Blay JY Caux C. Lymphopenia in cancer patients and its effects on response to immunotherapy: an opportunity for combination with cytokines? J Immunother Cancer. (2019) 7:85. 10.1186/s40425-019-0549-5 30922400 PMC6437964

[36] Asgari MM Ray GT Quesenberry CP Katz KA Silverberg MJ. Association of multiple primary skin cancers with human immunodeficiency virus infection, CD4 count, and viral load. JAMA Dermatol. (2017) 153:892–6. 10.1001/jamadermatol.2017.1716 28700773 PMC5710423

[37] Hernández-Ramírez RU Shiels MS Dubrow R Engels EA. Cancer risk in HIV-infected people in the USA from 1996 to 2012: a population-based, registry-linkage study. Lancet HIV. (2017) 4:e495–504. 10.1016/S2352-3018(17)30125-X 28803888 PMC5669995

[38] Ryom L Lundgren JD De Wit S Kovari H Reiss P Law M. Use of antiretroviral therapy and risk of end-stage liver disease and hepatocellular carcinoma in HIV-positive persons. AIDS. (2016) 30:1731–43. 10.1097/QAD.0000000000001018 26752282

[39] Yarchoan R Uldrick TS HIV-associated cancers and related diseases. N Engl J Med. (2018) 378:1029–41. 10.1056/NEJMra1615896 29539283 PMC6890231

[40] Ebogo-Belobo JT Kagoué Simeni LA Mbassa Nnouma G Lawan Loubou M Abamé I Tchuisseu Hapi A. Incidence of cancer in people living with HIV and prognostic value of current CD4. Bull Cancer. (2019) 106:201–5. 10.1016/j.bulcan.2018.11.003 30502923

[41] Dubrow R Qin L Lin H Hernández-Ramírez RU Neugebauer RS Leyden W. Association of CD4+ T-cell Count, HIV-1 RNA viral load, and antiretroviral therapy with kaposi sarcoma risk among HIV-infected persons in the United States and Canada. J Acquir Immune Defic Syndr. (2017) 75:382–90. 10.1097/QAI.0000000000001394 28394855 PMC5490794

[42] Shepherd L Ryom L Law M Hatleberg CI de Wit S Monforte AD. Differences in virological and immunological risk factors for non-Hodgkin and Hodgkin lymphoma. J Natl Cancer Inst. (2018) 110:598–607. 10.1093/jnci/djx249 29267895 PMC6693037

[43] Rohner E Bütikofer L Schmidlin K Sengayi M Maskew M Giddy J Comparison of Kaposi sarcoma risk in human immunodeficiency virus-positive adults across 5 continents: a multiregional multicohort study. Clin Infect Dis. (2017) 65:1316–26. 10.1093/cid/cix480 28531260 PMC5850623

[44] Ostroumov D Fekete-Drimusz N Saborowski M Kühnel F Woller N. CD4 and CD8 T lymphocyte interplay in controlling tumor growth. Cell Mol Life Sci. (2018) 75:689–713. 10.1007/s00018-017-2686-7 29032503 PMC5769828

[45] Serrano-Villar S Pérez-Elías MJ Dronda F Casado JL Moreno A Royuela A. Increased risk of serious non-AIDS-related events in HIV-infected subjects on antiretroviral therapy associated with a low CD4/CD8 ratio. PLoS ONE. (2014) 9:e85798. 10.1371/journal.pone.0085798 24497929 PMC3907380

[46] Serrano-Villar S Sainz T Lee SA Hunt PW Sinclair E Shacklett BL. HIV-infected individuals with low CD4/CD8 ratio despite effective antiretroviral therapy exhibit altered T cell subsets, heightened CD8+ T cell activation, and increased risk of non-AIDS morbidity and mortality. PLoS Pathog. (2014) 10:e1004078. 10.1371/journal.ppat.1004078 24831517 PMC4022662

[47] igel K Makinson A Thaler J Lung cancer in persons with HIV. Curr Opin HIV AIDS. (2017) 12:31–8. 10.1097/COH.0000000000000326 27607596 PMC5241551

[48] Clifford GM Rickenbach M Lise M Dal Maso L Battegay M Bohlius J. Hodgkin lymphoma in the Swiss HIV cohort study. Blood. (2009) 113:5737–42. 10.1182/blood-2009-02-204172 19336755

[49] Hema MN Ferry T Dupon M Cuzin L Verdon R Thiébaut R. Low CD4/CD8 ratio is associated with non AIDS-defining cancers in patients on antiretroviral therapy: ANRS CO8 (Aproco/Copilote) prospective cohort study. PLoS ONE. (2016) 11:e0161594. 10.1371/journal.pone.0161594 27548257 PMC4993515

[50] Okoye IS Houghton M Tyrrell L Barakat K Elahi S. Coinhibitory receptor expression and immune checkpoint blockade: maintaining a balance in CD8(+) T cell responses to chronic viral infections and cancer. Front Immunol. (2017) 8:1215. 10.3389/fimmu.2017.01215 29033936 PMC5626929

[51] Konopnicki D Manigart Y Gilles C Barlow P de Marchin J Feoli F. High-risk human papillomavirus infection in HIV-positive African women living in Europe. J Int AIDS Soc. (2013) 16:18023. 10.7448/IAS.16.1.18023 23406965 PMC3574170

[52] Luchters SM Vanden Broeck D Chersich MF Nel A Delva W Mandaliya K. Association of HIV infection with distribution and viral load of HPV types in Kenya: a survey with 820 female sex workers. BMC Infect Dis. (2010) 10:18. 10.1186/1471-2334-10-18 20102630 PMC2845133

[53] Massad LS Seaberg EC Wright RL Darragh T Lee YC Colie C. Squamous cervical lesions in women with human immunodeficiency virus. Obstet Gynecol. (2008) 111:1388–93. 10.1097/AOG.0b013e3181744619 18515523

[54] Abraham SN Miao Y. The nature of immune responses to urinary tract infections. Nat Rev Immunol. (2015) 15:655–63. 10.1038/nri3887 26388331 PMC4926313

[55] Mugo NR Eckert L Magaret AS Cheng A Mwaniki L Ngure K. Quadrivalent HPV vaccine in HIV-1-infected early adolescent girls and boys in Kenya: Month 7 and 12 post vaccine immunogenicity and correlation with immune status. Vaccine. (2018) 36:7025–32. 10.1016/j.vaccine.2018.09.059 30297124

[56] Scherer EM Smith RA Simonich CA Niyonzima N Carter JJ Galloway DA. Characteristics of memory B cells elicited by a highly efficacious HPV vaccine in subjects with no pre-existing immunity. PLoS Pathog. (2014) 10:e1004461. 10.1371/journal.ppat.1004461 25330199 PMC4199765

[57] Kenter GG Welters MJ Valentijn AR Lowik MJ Berends-van der Meer DM Vloon AP. Vaccination against HPV-16 oncoproteins for vulvar intraepithelial neoplasia. N Engl J Med. (2009) 361:1838–47. 10.1056/NEJMoa0810097 19890126

[58] Maldonado L Teague JE Morrow MP Jotova I Wu TC Wang C. Intramuscular therapeutic vaccination targeting HPV16 induces T cell responses that localize in mucosal lesions. Sci Transl Med. (2014) 6:221ra13. 10.1126/scitranslmed.3007323 24477000 PMC4086631

[59] Saeidi A Zandi K Cheok YY Saeidi H Wong WF Lee CYQ. T-cell exhaustion in chronic infections: reversing the state of exhaustion and reinvigorating optimal protective immune responses. Front Immunol. (2018) 9:2569. 10.3389/fimmu.2018.02569 30473697 PMC6237934

[60] Unemori P Leslie KS Hunt PW Sinclair E Epling L Mitsuyasu R. Immunosenescence is associated with presence of Kaposi's sarcoma in antiretroviral treated HIV infection. AIDS. (2013) 27:1735–42. 10.1097/QAD.0b013e3283601144 23435301 PMC4063793

[61] Hernández DM Valderrama S Gualtero S Hernández C López M Herrera MV. Loss of T-cell multifunctionality and TCR-Vβ repertoire against Epstein- Barr virus is associated with worse prognosis and clinical parameters in HIV+patients. Front Immunol. (2018) 9:2291. 10.3389/fimmu.2018.02291 30337929 PMC6180205

[62] Zheng J Wang L Cheng Z Pei Z Zhang Z Li Z. Molecular changes of lung malignancy in HIV infection. Sci Rep. (2018) 8:13128. 10.1038/s41598-018-31572-6 30177858 PMC6120915

[63] Bender Ignacio RA Lin LL Rajdev L Chiao E. Evolving paradigms in HIV malignancies: review of ongoing clinical trials. J Natl Compr Canc Netw. (2018) 16:1018–26. 10.6004/jnccn.2018.7064 30099376 PMC6109631

[64] Mocroft A Kirk O Clumeck N Gargalianos-Kakolyris P Trocha H Chentsova N. The changing pattern of Kaposi sarcoma in patients with HIV, 1994–2003: the EuroSIDA Study. Cancer. (2004) 100:2644–54. 10.1002/cncr.20309 15197808

[65] Franceschi S Maso LD Rickenbach M Polesel J Hirschel B Cavassini M. Kaposi sarcoma incidence in the Swiss HIV Cohort Study before and after highly active antiretroviral therapy. Brit J Cancer. (2008) 99:800–4. 10.1038/sj.bjc.6604520 18665172 PMC2528138

[66] Gallafent JH Buskin SE De Turk PB Aboulafia DM Gallafent JH Buskin SE. Profile of patients with Kaposi's sarcoma in the era of highly active antiretroviral therapy. J Clin Oncol. (2005) 23:1253–60. 10.1200/JCO.2005.04.156 15718323

[67] Antman K Chang Y. Kaposi's sarcoma. N Engl J Med. (2000) 342:1027–38. 10.1056/NEJM200004063421407 10749966

[68] Heppt MV Schlaak M Eigentler TK Kähler KC Kiecker F Loquai C. Checkpoint blockade for metastatic melanoma and Merkel cell carcinoma in HIV-positive patients. Ann Oncol. (2017):3104–6. 10.1093/annonc/mdx538 28950303

[69] Tio M Rai R Ezeoke OM Zimmer L Khoo C Park JJ Anti-PD-1/PD-L1 immunotherapy in patients with solid organ transplant, HIV or hepatitis B/C infection. Eur J Cancer. (2018) 104:137–44. 10.1016/j.ejca.2018.09.017 30347289 PMC10176037

[70] Lavolé A Guihot A Veyri M Lambotte O Autran B Cloarec N PD-1 blockade in HIV-infected patients with lung cancer: a new challenge or already a strategy? Ann Oncol. (2018) 29:1065–6. 10.1093/annonc/mdx817 29346600

[71] Opdivo (nivolumab). Princeton, NJ: Bristol-Meyers Squibb Company (2018).

[72] Keytruda (pembrolizumab). Whitehouse Station, NJ: Merck & Company (2016).

[73] Polizzotto MN Uldrick TS Wyvill KM Aleman K Peer CJ Bevans M. Pomalidomide for Symptomatic Kaposi's Sarcoma in People With and Without HIV Infection: a phase I/II study. J Clin Oncol. (2016) 34:4125–31. 10.1200/JCO.2016.69.3812 27863194 PMC5477825

[74] Polizzotto MN Uldrick T Wyvill KM Aleman K Peer CJ Bevans M Pomalidomide induces expansion of activated and central memory CD4+ and CD8+ T cells *in vivo* in patients with and without HIV infection. Blood. (2014) 124:4128–31. 10.1200/JCO.2016.69.3812t

[75] Pomalyst (pomalidomide). Summit, NJ: Celgene Corporation (2017).

[76] Wang CC Thanh C Gibson EA Ball-Burack M Hogan LE Descours B. Transient loss of detectable HIV-1 RNA following brentuximab vedotin anti-CD30 therapy for Hodgkin lymphoma. Blood Adv. (2018) 2:3479–82. 10.1182/bloodadvances.2018024364 30530753 PMC6290106

[77] Adcetris (brentuximab vedotin). Bothell, WA: Seattle Genetics, Inc (2014).

[78] Ruxrungtham K Sirivichayakul S Buranapraditkun S Krause W. Alemtuzumab-induced elimination of HIV-1-infected immune cells. J Virus Erad. (2016) 2:12–8. 27482429 10.1016/S2055-6640(20)30694-4 PMC4946689

[79] Rasmussen TA McMahon J Chang JJ Symons J Roche M Dantanarayana A. Impact of alemtuzumab on HIV persistence in an HIV-infected individual on antiretroviral therapy with Sezary syndrome. AIDS. (2017) 31:1839–45. 10.1097/QAD.0000000000001540 28514279 PMC5990417

[80] Campath (alemtuzumab). Cambridge, MA: Millennium and ILEX Partners, LP (2001).

[81] Guarnera C Bramanti P Mazzon E. Alemtuzumab: a review of efficacy and risks in the treatment of relapsing remitting multiple sclerosis. Ther Clin Risk Manag. (2017) 13:871–9. 10.2147/TCRM.S134398 28761351 PMC5522829

[82] Mackall CL Fry TJ Gress RE. Harnessing the biology of IL-7 for therapeutic application. Nat Rev Immunol. (2011) 11:330–42. 10.1038/nri2970 21508983 PMC7351348

[83] Wang C Edilova MI Wagar LE Mujib S Singer M Bernard NF. Effect of IL-7 Therapy on phospho-ribosomal protein S6 and TRAF1 expression in HIV-specific CD8 T cells in patients receiving antiretroviral therapy. J Immunol. (2018) 200:558–64. 10.4049/jimmunol.1601254 29222166 PMC5760332

[84] Logerot S Rancez M Charmeteau-de Muylder B Figueiredo-Morgado S Rozlan S Tambussi G. HIV reservoir dynamics in HAART-treated poor immunological responder patients under IL-7 therapy. AIDS. (2018) 32:715–20. 10.1097/QAD.0000000000001752 29369157

[85] Fry TJ Connick E Falloon J Lederman MM Liewehr DJ Spritzler J. A potential role for interleukin-7 in T-cell homeostasis. Blood. (2001) 97:2983–90. 10.1182/blood.V97.10.2983 11342421

[86] Managlia EZ Landay A Al-Harthi L. Interleukin-7 induces HIV replication in primary naive T cells through a nuclear factor of activated T cell (NFAT)-dependent pathway. Virology. (2006) 350:443–52. 10.1016/j.virol.2006.02.019 16542695

[87] Chahroudi A Silvestri G. Interleukin-7 in HIV pathogenesis and therapy. Eur Cytokine Netw. (2010) 21:202–7. 10.1684/ecn.2010.0205 20729180

[88] Le Saout C Luckey MA Villarino AV Smith M Hasley RB Myers TG. IL-7-dependent STAT1 activation limits homeostatic CD4+ T cell expansion. JCI insight (2017) 2:96228. 10.1172/jci.insight.96228 29202461 PMC5752389

[89] Ponchel F Cuthbert RJ Goëb V. IL-7 and lymphopenia. Clin Chim Acta. (2011) 412:7–16. 10.1016/j.cca.2010.09.002 20850425

[90] Sportès C Hakim FT Memon SA Zhang H Chua KS Brown MR. Administration of rhIL-7 in humans increases *in vivo* TCR repertoire diversity by preferential expansion of naive T cell subsets. J Exp Med. (2008) 205:1701–14. 10.1084/jem.20071681 18573906 PMC2442646

[91] Brunda MJ. Interleukin-12. J Leukoc Biol. (1994) 55:280–8. 10.1002/jlb.55.2.280 7905508

[92] Little RF Pluda JM Wyvill KM Rodriguez-Chavez IR Tosato G Catanzaro AT. Activity of subcutaneous interleukin-12 in AIDS-related Kaposi sarcoma. Blood. (2006) 107:4650–7. 10.1182/blood-2005-11-4455 16507779 PMC1475826

[93] Vandergeeten C Fromentin R Chomont N. The role of cytokines in the establishment, persistence and eradication of the HIV reservoir. Cytokine Growth Factor Rev (2012) 23:143–9. 10.1016/j.cytogfr.2012.05.001 22743037 PMC3767481

[94] Berard M Brandt K Bulfone-Paus S Tough DF. IL-15 promotes the survival of naive and memory phenotype CD8+ T cells. J Immunol. (2003) 170:5018–26. 10.4049/jimmunol.170.10.5018 12734346

[95] Mueller YM Katsikis PD IL-15 in HIV infection: pathogenic or therapeutic potential? Eur Cytokine Netw. (2010) 21:219–21. 20719708 10.1684/ecn.2010.0198

[96] Garrido C Abad-Fernandez M Tuyishime M Pollara JJ Ferrari G Soriano-Sarabia N. Interleukin-15-stimulated natural killer cells clear HIV-1-infected cells following latency reversal *ex vivo*. J Virol. (2018) 92:e00235–18. 10.1128/JVI.00235-18 29593039 PMC5974478

[97] Xu A Bhanumathy KK Wu J Ye Z Freywald A Leary SC. IL-15 signaling promotes adoptive effector T-cell survival and memory formation in irradiation-induced lymphopenia. Cell Biosci (2016) 6:30. 10.1186/s13578-016-0098-2 27158441 PMC4858849

[98] Krown SE Li P Von Roenn JH Paredes J Huang J Testa MA. Efficacy of low-dose interferon with antiretroviral therapy in Kaposi's sarcoma: a randomized phase II AIDS clinical trials group study. J Interferon Cytokine Res. (2002) 22:295–303. 10.1089/107999002753675712 12034036

[99] Shepherd FA Beaulieu R Gelmon K Thuot CA Sawka C Read S. Prospective randomized trial of two dose levels of interferon alfa with zidovudine for the treatment of Kaposi's sarcoma associated with human immunodeficiency virus infection: a Canadian HIV Clinical Trials Network study. J Clin Oncol. (1998) 16:1736–42. 10.1200/JCO.1998.16.5.1736 9586886

[100] Krown SE Gold JW Niedzwiecki D Bundow D Flomenberg N Gansbacher B. Interferon-alpha with zidovudine: safety, tolerance, and clinical and virologic effects in patients with Kaposi sarcoma associated with the acquired immunodeficiency syndrome (AIDS). Ann Inten Med. (1990) 112:812–21. 10.7326/0003-4819-112-11-812 1971504

[101] Strauss J Heery CR Kim JW Jochems C Donahue RN Montgomery AS. First-in-human phase i trial of a tumor-targeted cytokine (NHS-IL12) in subjects with metastatic solid tumors. Clin Cancer Res. (2019) 25:99–109. 10.1158/1078-0432.CCR-18-1512 30131389 PMC6320276

[102] Pfreundschuh M Kuhnt E Trümper L Osterborg A Trneny M Shepherd L. CHOP-like chemotherapy with or without rituximab in young patients with good-prognosis diffuse large-B-cell lymphoma: 6-year results of an open-label randomised study of the MabThera International Trial (MInT) Group. Lancet Oncol. (2011) 12:1013–22. 10.1016/S1470-2045(11)70235-2 21940214

[103] Coiffier B Thieblemont C Van Den Neste E Lepeu G Plantier I Castaigne S. Long-term outcome of patients in the LNH-98.5 trial, the first randomized study comparing rituximab-CHOP to standard CHOP chemotherapy in DLBCL patients: a study by the Groupe d'Etudes des Lymphomes de l'Adulte. Blood. (2010) 116:2040–5. 10.1182/blood-2010-03-276246 20548096 PMC2951853

[104] Coiffier B Lepage E Briere J Herbrecht R Tilly H Bouabdallah R. CHOP chemotherapy plus rituximab compared with CHOP alone in elderly patients with diffuse large-B-cell lymphoma. N Engl J Med. (2002) 346:235–42. 10.1056/NEJMoa011795 11807147

[105] Barta SK Xue X Wang D Tamari R Lee JY Mounier N. Treatment factors affecting outcomes in HIV-associated non-Hodgkin lymphomas: a pooled analysis of 1546 patients. Blood. (2013) 122:3251–62. 10.1182/blood-2013-04-498964 24014242 PMC3821722

[106] Rubinstein PG Moore PC Rudek MA Henry DH Ramos JC Ratner L. Brentuximab vedotin with AVD shows safety, in the absence of strong CYP3A4 inhibitors, in newly diagnosed HIV-associated Hodgkin lymphoma. AIDS. (2018) 32:605–11. 10.1097/QAD.0000000000001729 29280762 PMC5832596

[107] Sundar R Cho BC Brahmer JR Soo RA. Nivolumab in NSCLC: latest evidence and clinical potential. Ther Adv Med Oncol. (2015) 7:85–96. 10.1177/1758834014567470 25755681 PMC4346216

[108] Brahmer JR Tykodi SS Chow LQ Hwu WJ Topalian SL Hwu P. Safety and activity of anti–PD-L1 antibody in patients with advanced cancer. N Engl J Med. (2012) 366:2455–65. 10.1056/NEJMoa1200694 22658128 PMC3563263

[109] Uldrick TS Ison G Rudek MA Noy A Schwartz K Bruinooge S. Modernizing clinical trial eligibility criteria: recommendations of the american society of clinical oncology-friends of cancer research HIV working group. J Clin Oncol. (2017) 35:3774–80. 10.1200/JCO.2017.73.7338 28968173 PMC5793223

[110] Sandoval-Sus JD Mogollon-Duffo F Patel A Visweshwar N Laber DA Kim R. Nivolumab as salvage treatment in a patient with HIV-related relapsed/refractory Hodgkin lymphoma and liver failure with encephalopathy. J Immunother Cancer. (2017) 5:49. 10.1186/s40425-017-0252-3 28642818 PMC5477132

[111] Davar D Wilson M Pruckner C Kirkwood JM PD-1 blockade in advanced melanoma in patients with hepatitis C and/or HIV. Case Rep Oncol Med. (2015) 2015:737389 10.1155/2015/737389 26448890 PMC4581502

[112] Husnain M Park W Ramos JC Johnson TE Chan J Dasari A. Complete response to ipilimumab and nivolumab therapy in a patient with extensive extrapulmonary high-grade small cell carcinoma of the pancreas and HIV infection. J Immunother Cancer. (2018) 6:66. 10.1186/s40425-018-0379-x 29986769 PMC6036694

[113] Burke MM Kluger HM Golden M Heller KN Hoos A Sznol M. Case Report: response to ipilimumab in a patient with HIV with metastatic melanoma. J ClinOncol. (2011) 29:e792–4. 10.1200/JCO.2011.36.9199 21990407

[114] Spano JP Veyri M Gobert A Guihot A Perré P Kerjouan M Immunotherapy for cancer in people living with HIV: safety with an efficacy signal from the series in real life experience on behalf of the French CANCERVIH network. AIDS. (2019) 33:F13–9. 10.1097/QAD.0000000000002298 31259762

[115] Chang E Sabichi AL Kramer JR Hartman C Royse KE White DL. Nivolumab treatment for cancers in the HIV-infected population. J Immunother. (2018) 41:379–83. 10.1097/CJI.0000000000000240 30020193 PMC6128753

[116] Evans VA van der Sluis RM Solomon A Dantanarayana A McNeil C Garsia R. Programmed cell death-1 contributes to the establishment and maintenance of HIV-1 latency. AIDS. (2018) 32:1491–7. 10.1097/QAD.0000000000001849 29746296 PMC6026054

[117] Cook MR Kim C. Safety and efficacy of immune checkpoint inhibitor therapy in patients with HIV infection and advanced-stage cancer: a systematic review. JAMA Oncol. (2019) 5:1049–54. 10.1001/jamaoncol.2018.6737 30730549

[118] Uldrick TS Gonçalves PH Abdul-Hay M Claeys AJ Emu B Ernstoff MS. Assessment of the safety of pembrolizumab in patients with HIV and advanced cancer-A phase 1 study. JAMA Oncol. (2019). [Epub ahead of print]. 10.1001/jamaoncol.2019.2244 31154457 PMC6547135

[119] González-Cao M Moran T Dalmau J Garcia-Corbacho J Bernabé R Juan O Phase II study of durvalumab (MEDI4736) in cancer patients HIV-1-infected. J Clin Oncol. (2019) 37:2501 10.1200/JCO.2019.37.15_suppl.2501

[120] Finzi D Hermankova M Pierson T Carruth LM Buck C Chaisson RE. Identification of a reservoir for HIV-1 in patients on highly active antiretroviral therapy. Science. (1997) 278:1295–300. 10.1126/science.278.5341.1295 9360927

[121] Wong JK Hezareh M Günthard HF Havlir DV Ignacio CC Spina CA. Recovery of replication-competent HIV despite prolonged suppression of plasma viremia. Science. (1997) 278:1291–5. 10.1126/science.278.5341.1291 9360926

[122] Chun TW Stuyver L Mizell SB Ehler LA Mican JA Baseler M. Presence of an inducible HIV-1 latent reservoir during highly active antiretroviral therapy. Proc Natl Acad Sci USA. (1997) 94:13193–7. 10.1073/pnas.94.24.13193 9371822 PMC24285

[123] Simonetti FR Sobolewski MD Fyne E Shao W Spindler J Hattori J. Clonally expanded CD4+ T cells can produce infectious HIV-1 *in vivo*. Proc Natl Acad Sci USA. (2016) 113:1883–8. 10.1073/pnas.1522675113 26858442 PMC4763755

[124] Wagner TA McLaughlin S Garg K Cheung CYK Larsen BB Styrchak S. HIV latency. Proliferation of cells with HIV integrated into cancer genes contributes to persistent infection. Science. (2014) 345:570–3. 10.1126/science.1256304 25011556 PMC4230336

[125] Schröder ARW Shinn P Chen H Berry C Ecker JR Bushman F. HIV-1 integration in the human genome favors active genes and local hotspots. Cell. (2002) 110:521–9. 10.1016/S0092-8674(02)00864-4 12202041

[126] Haworth KG Schefter LE Norgaard ZK Ironside C Adair JE Kiem HP. HIV infection results in clonal expansions containing integrations within pathogenesis-related biological pathways. JCI insight. (2018) 3:99127. 10.1172/jci.insight.99127 29997284 PMC6124524

[127] Maldarelli F Wu X Su L Simonetti FR Shao W Hill S. HIV latency. Specific HIV integration sites are linked to clonal expansion and persistence of infected cells. Science. (2014) 345:179–83. 10.1126/science.1254194 24968937 PMC4262401

[128] Wightman F Solomon A Kumar SS Urriola N Gallagher K Hiener B. Effect of ipilimumab on the HIV reservoir in an HIV-infected individual with metastatic melanoma. AIDS. (2015) 29:504–6. 10.1097/QAD.0000000000000562 25628259 PMC4492799

[129] Offersen R Nissen SK Rasmussen TA Østergaard L Denton PW Søgaard OS Pembrozlizumab Induces HIV latency reversal in HIV+ Individuals on ART with Cancer. In: Conference on Retroviruses and Opportunistic Infections (CROI). Seattle, WA (2019). p. Abstract 27.

[130] Hentrich M Schipek-Voigt K Jäger H Schulz S Schmid P Stötzer O. Nivolumab in HIV-related non-small-cell lung cancer. Ann Oncol. (2017) 28:2890. 10.1093/annonc/mdx321 29106466

[131] McCullar B Alloway T Martin M. Durable complete response to nivolumab in a patient with HIV and metastatic non-small cell lung cancer. J Thorac Dis. (2017) 9:E540–2. 10.21037/jtd.2017.05.32 28740692 PMC5506156

[132] Le Garff G Samri A Lambert-Niclot S Even S Lavolé A Cadranel J. Transient HIV-specific T cells increase and inflammation in an HIV-infected patient treated with nivolumab. AIDS. (2017) 31:1048–51. 10.1097/QAD.0000000000001429 28350581

[133] Macatangay BJ Rinaldo CR. PD-1 blockade: a promising immunotherapy for HIV? Cell Sci. (2009) 5:61–5. 20490361 PMC2872789

[134] Chomont N El-Far M Ancuta P Trautmann L Procopio FA Yassine-Diab B. HIV reservoir size and persistence are driven by T cell survival and homeostatic proliferation. Nat Med. (2009) 15:893–900. 10.1038/nm.1972 19543283 PMC2859814

[135] Fromentin R DaFonseca S Costiniuk CT El-Far M Procopio FA Hecht FM. PD-1 blockade potentiates HIV latency reversal ex vivo in CD4(+) T cells from ART-suppressed individuals. Nat Commun. (2019) 10:814. 10.1038/s41467-019-08798-7 30778080 PMC6379401

[136] Banga R Procopio FA Noto A Pollakis G Cavassini M Ohmiti K. PD-1(+) and follicular helper T cells are responsible for persistent HIV-1 transcription in treated aviremic individuals. Nat Med. (2016) 22:754–61. 10.1038/nm.4113 27239760

[137] Guihot A Marcelin AG Massiani MA Samri A Soulié C Autran B. Drastic decrease of the HIV reservoir in a patient treated with nivolumab for lung cancer. Ann Oncol. (2018) 29:517–8. 10.1093/annonc/mdx696 29206889

[138] Colston E Grasela D Gardiner D Bucy RP Vakkalagadda B Korman AJ. An open-label, multiple ascending dose study of the anti-CTLA-4 antibody ipilimumab in viremic HIV patients. PLoS ONE. (2018) 13:e0198158. 10.1371/journal.pone.0198158 29879143 PMC5991705

[139] Biswas P Smith CA Goletti D Hardy EC Jackson RW Fauci AS. Cross-linking of CD30 induces HIV expression in chronically infected T cells. Immunity. (1995) 2:587–96. 10.1016/1074-7613(95)90003-9 7540942

[140] Hogan LE Vasquez J Hobbs KS Hanhauser E Aguilar-Rodriguez B Hussien R. Increased HIV-1 transcriptional activity and infectious burden in peripheral blood and gut-associated CD4+ T cells expressing CD30. PLoS Pathog. (2018) 14:e1006856. 10.1371/journal.ppat.1006856 29470552 PMC5823470

[141] Gupta RK Abdul-Jawad S McCoy LE Mok HP Peppa D Salgado M. HIV-1 remission following CCR5Delta32/Delta32 haematopoietic stem-cell transplantation. Nature. (2019) 568:244–8. 10.1038/s41586-019-1027-4 30836379 PMC7275870

[142] Hodge JN Srinivasula S Hu Z Read SW Porter BO Kim I. Decreases in IL-7 levels during antiretroviral treatment of HIV infection suggest a primary mechanism of receptor-mediated clearance. Blood. (2011) 118:3244–53. 10.1182/blood-2010-12-323600 21778338 PMC3179394

[143] Lévy Y Sereti I Tambussi G Routy JP Lelièvre JD Delfraissy JF. Effects of recombinant human interleukin 7 on T-cell recovery and thymic output in HIV-infected patients receiving antiretroviral therapy: results of a phase I/IIa randomized, placebo-controlled, multicenter study. Clin Infect Dis. (2012) 55:291–300. 10.1093/cid/cis383 22550117 PMC3381639

[144] Mueller YM Bojczuk PM Halstead ES Kim AH Witek J Altman JD. IL-15 enhances survival and function of HIV-specific CD8+ T cells. Blood. (2003) 101:1024–9. 10.1182/blood-2002-07-1957 12393488

[145] Hasan MS Kallas EG Thomas EK Looney J Campbell M Evans TG. Effects of interleukin-15 on in vitro human T cell proliferation and activation. J Interferon Cytokine Res. (2000) 20:119–23. 10.1089/107999000312513 10714546

[146] Hütter G Nowak D Mossner M Ganepola S Müssig A Allers K. Long-term control of HIV by CCR5 Delta32/Delta32 stem-cell transplantation. N Engl J Med. (2009) 360:692–8. 10.1056/NEJMoa0802905 19213682

[147] Hutter G Ganepola S. Eradication of HIV by transplantation of CCR5-deficient hematopoietic stem cells. Sci World J. (2011) 11:1068–76. 10.1100/tsw.2011.102 21552772 PMC5720062

[148] Salgado M Kwon M Gálvez C Badiola J Nijhuis M Bandera A. Mechanisms that contribute to a profound reduction of the HIV-1 reservoir after allogeneic stem cell transplant. Ann Intern Med. (2018) 169:674–83. 10.7326/M18-0759 30326031

[149] Henrich TJ Hanhauser E Marty FM Sirignano MN Keating S Lee TH. Antiretroviral-free HIV-1 remission and viral rebound after allogeneic stem cell transplantation: report of 2 cases. Ann Intern Med. (2014) 161:319–27. 10.7326/M14-1027 25047577 PMC4236912

[150] Macpherson JL Boyd MP Arndt AJ Todd AV Fanning GC Ely JA. Long-term survival and concomitant gene expression of ribozyme-transduced CD4+ T-lymphocytes in HIV-infected patients. J Gene Med. (2005) 7:552–64. 10.1002/jgm.705 15655805

[151] Mitsuyasu RT Merigan TC Carr A Zack JA Winters MA Workman C. Phase 2 gene therapy trial of an anti-HIV ribozyme in autologous CD34+ cells. Nat Med. (2009) 15:285–92. 10.1038/nm.1932 19219022 PMC2768566

[152] Burnett JC Zaia JA Rossi JJ. Creating genetic resistance to HIV. Curr Opin Immunol. (2012) 24:625–32. 10.1016/j.coi.2012.08.013 22985479 PMC3478429

[153] Durand CM Blankson JN Siliciano RF. Developing strategies for HIV-1 eradication. Trends Immunol. (2012) 33:554–62. 10.1016/j.it.2012.07.001 22867874 PMC3963166

[154] Persad GC Little RF Grady C. Including persons with HIV infection in cancer clinical trials. J Clin Oncol. (2008) 26:1027–32. 10.1200/JCO.2007.14.5532 18309938

